# Supplementary material for: Human TH17 cells engage gasdermin E pores to release IL-1α on NLRP3 inflammasome activation
Source: Nat Immunol. 2023 Jan 5;24(2):295–308. doi: 10.1038/s41590-022-01386-w (PMC9892007; doi:10.1038/s41590-022-01386-w)
Supplement: Supplementary file 1 — Supplementary Figs. 1–11 with legends. [file 41590_2022_1386_MOESM1_ESM.pdf]

nature immunology

Article

https://doi.org/10.1038/s41590-022-01386-w

Human T_H17 cells engage gasdermin E pores to release IL-1α on NLRP3 inflammasome activation

In the format provided by the authors and unedited

Supplementary Material

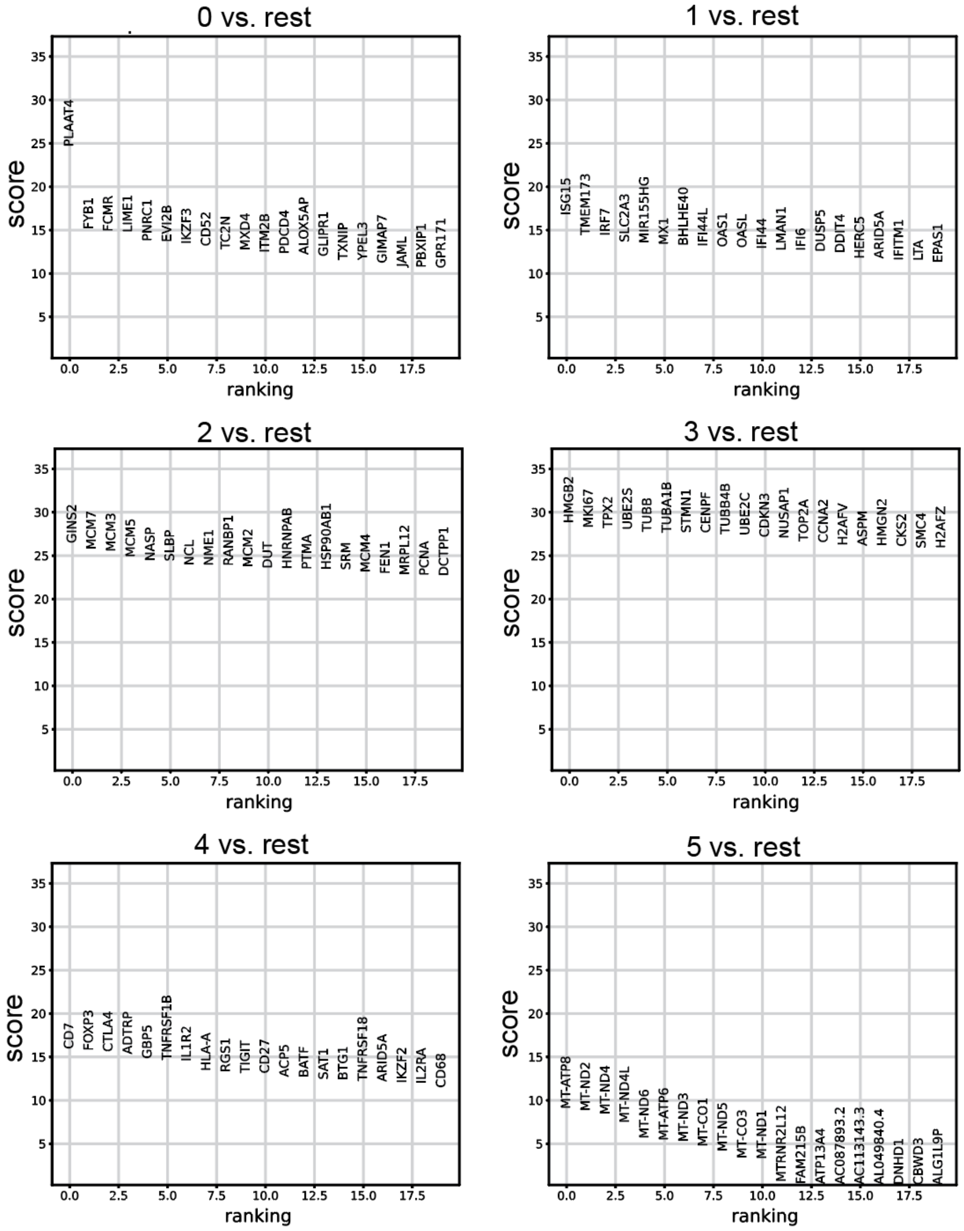

Supplementary Fig. 1. Top upregulated genes in Leiden clusters of the human Th17-cell population. scRNAseq and Leiden clustering of human Th17 cells stimulated for 5 days with anti-CD3 and anti-CD28 mAbs. Shown are the top 20 upregulated genes in individual Leiden clusters (0-5) as determined by Wilcoxon-rank-sum test with Benjamini-Hochberg correction for multiple testing as implemented in scanpy. The genes are ranked by the z score underlying the p value computation.

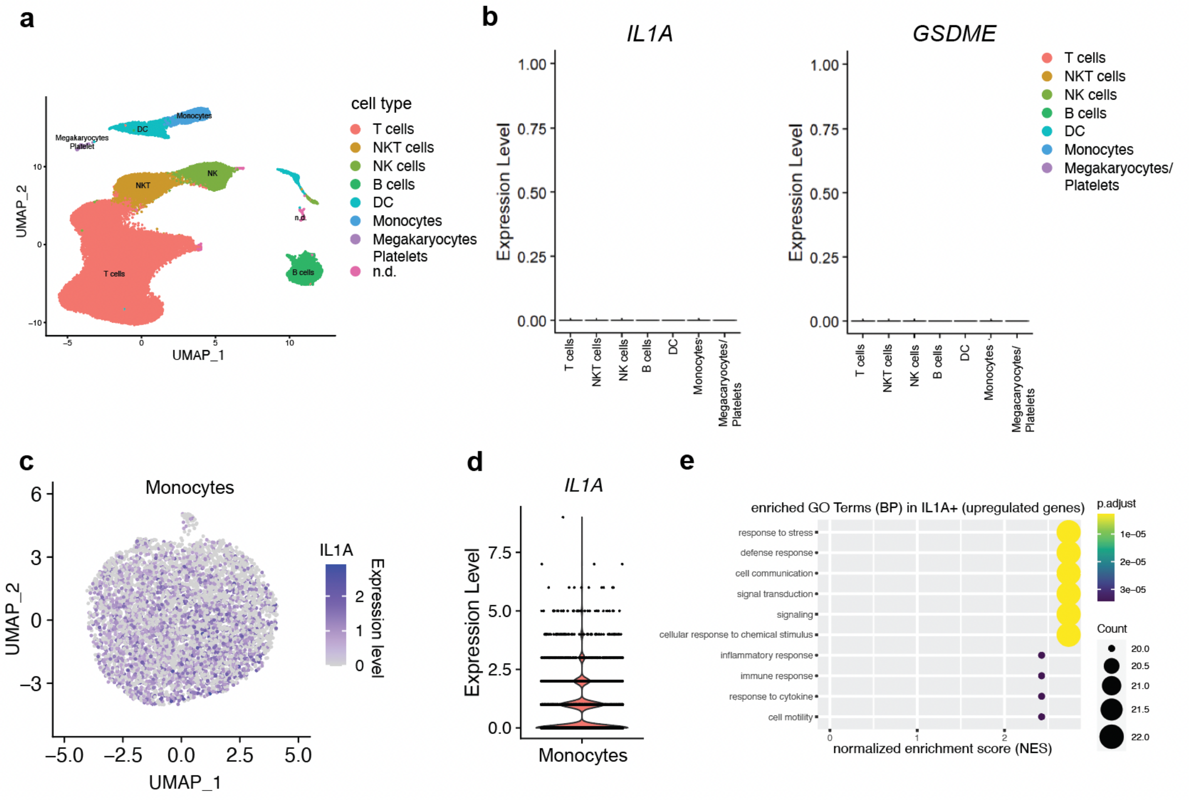

Supplementary Fig. 2. Differential expression of *IL1A* or *GSDME* in distinct immune cell populations from the peripheral blood. a, UMAP of PBMCs (68K) (public dataset from 10x Genomics) with Louvain clustering and cell type annotation by marker genes (Seurat, version 4.1.0). **b,** Violin plots with cell types identified in (a) showing the absence of *IL1A* and *GSDME* gene expression. **c, d**, UMAP (c) and violin plot (d) with analysis of *IL1A* expression levels in CD14^{+} monocytes treated with LPS for 6 h before scRNA-seq as described previously (GSE159113). **e,** Enrichment analysis for unbiased GO terms in clusterProfiler by performing one-sided Fisher's exact test with Benjamini-Hochberg correction with p-value cutoff set at 0.05 for genes differentially expressed between *IL1A*$^{+}$ and *IL1A*$^{−}$ monocytes after LPS stimulation.

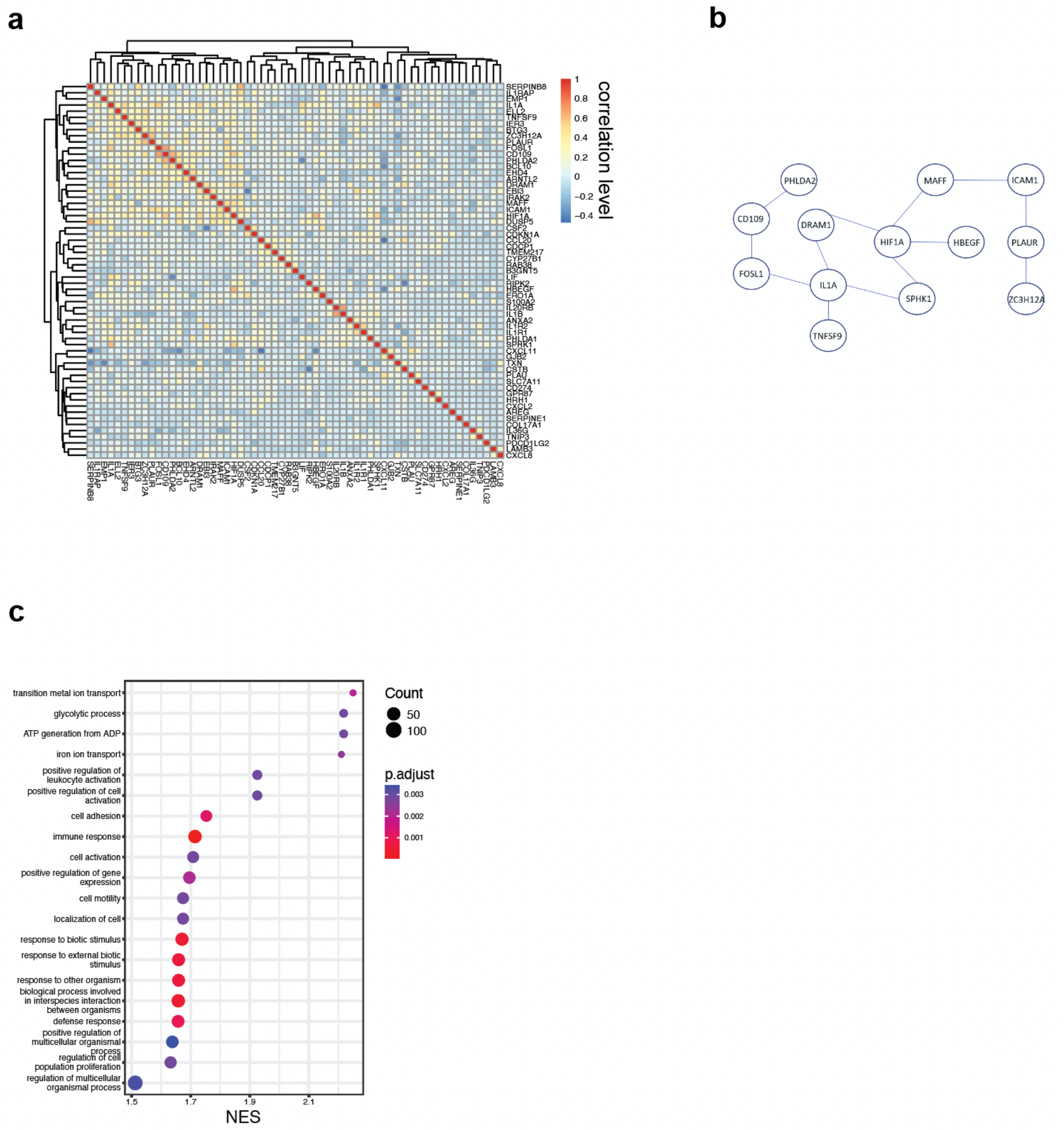

Supplementary Fig. 3. *IL1A* gene coexpression network for human Th17 cells. a, Heatmap showing correlation of *IL1A* coexpression by human Th17 cells (range: -0.4 to 1) with genes from the ARCH4 database that lists the top 100 genes known to be coexpressed with *IL1A* by interrogation of multiple publicly available transcriptomic datasets. Shown is the correlation value between gene pairs computed with scLink. **b,** Network plotted using the igraph R-package (version 1.3.4) shows 13 genes, which are directly or indirectly co-expressed with *IL1A* in Th17 cells based on a correlation value > 0.5 computed with scLink. **c,** Enrichment analysis with GO terms for *IL1A* coexpressing genes using clusterProfiler, showing top 20 resulting enrichment terms and the adjusted p-values. NES: Normalized Enrichment Score.

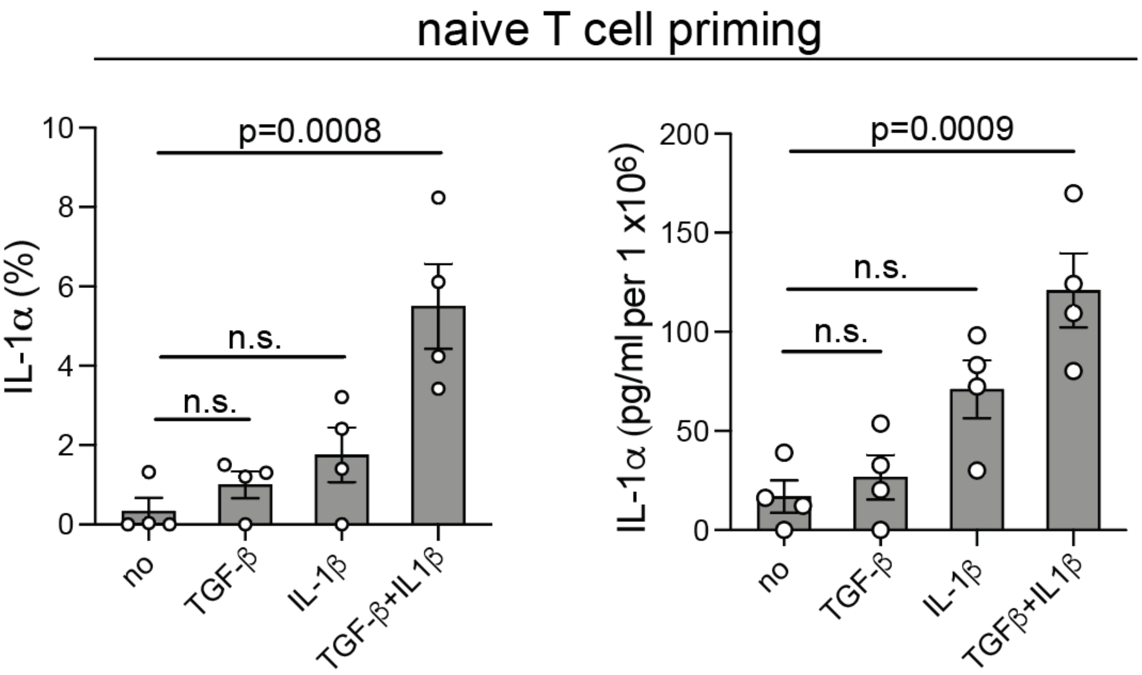

Supplementary Fig. 4. Synergistic activity of the Th17-cell priming cytokines IL-1β and TGF-β for IL-1α induction. Naïve Th cells were stimulated with anti-CD3 and anti-CD28 mAbs for 5 days before intracellular cytokine staining and flow cytometric analysis (left) or ELISA analysis (right). One-way ANOVA. Each circle indicates an individual healthy blood donor (n=4 biologically independent samples examined over 2 independent experiments). Data are presented as mean ± SEM.

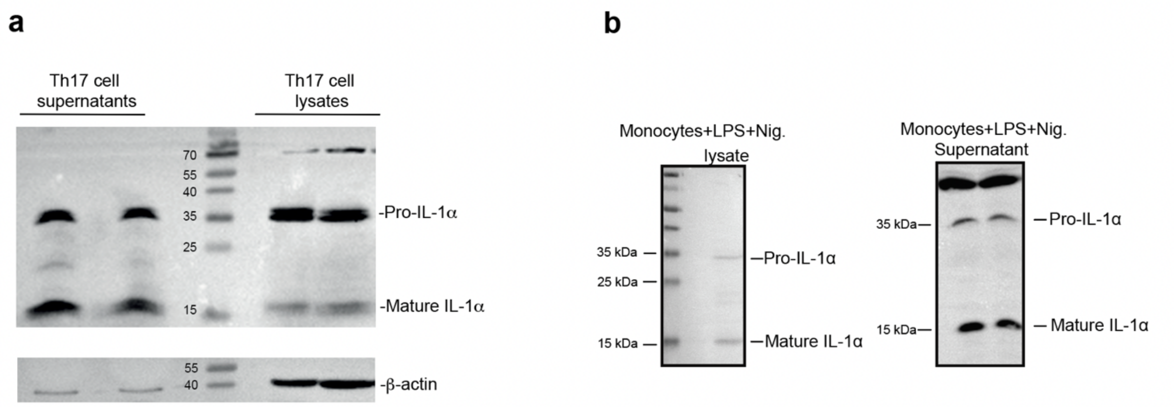

Supplementary Fig. 5. Distribution of pro-forms and mature forms of IL-1α in the supernatant and intracellularly in Th17 cells versus monocytes. a, Western blot analysis of cell culture supernatants and cell lysates from human Th17 cells stimulated for 5 days with anti-CD3 and anti-CD28 mAbs. One experiment with two independent blood donors is shown. **b,** Western blot analysis of cell lysates (left) and cell culture supernatants (right) from $CD14^{+}$ monocytes stimulated for 24 h with LPS and for 30 min with nigericin (Nig.) for comparison to Th17 cells as shown in Fig. 3a. The results of representative experiments are shown (n=3 biologically independent samples).

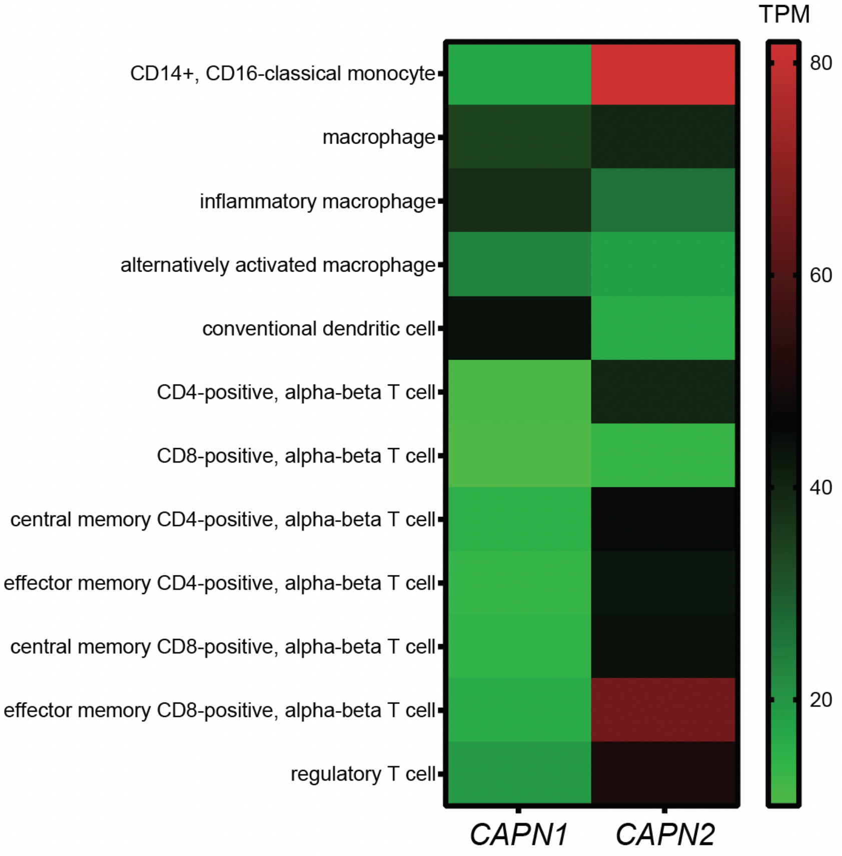

Supplementary Fig. 6. Differential expression of *CAPN1* and *CAPN2* in different immune cell types. Strand-specific RNA-seq of rRNA-depleted total RNA from cultured or uncultured primary cells of different hematopoietic lineages from healthy individuals in the BLUEPRINTepigenome project. A heat map of differential gene expression is shown. Public data were obtained from EMBL-EBI (https://www.ebi.ac.uk). *CAPN1*, calpain 1; *CAPN2*, calpain 2**.**

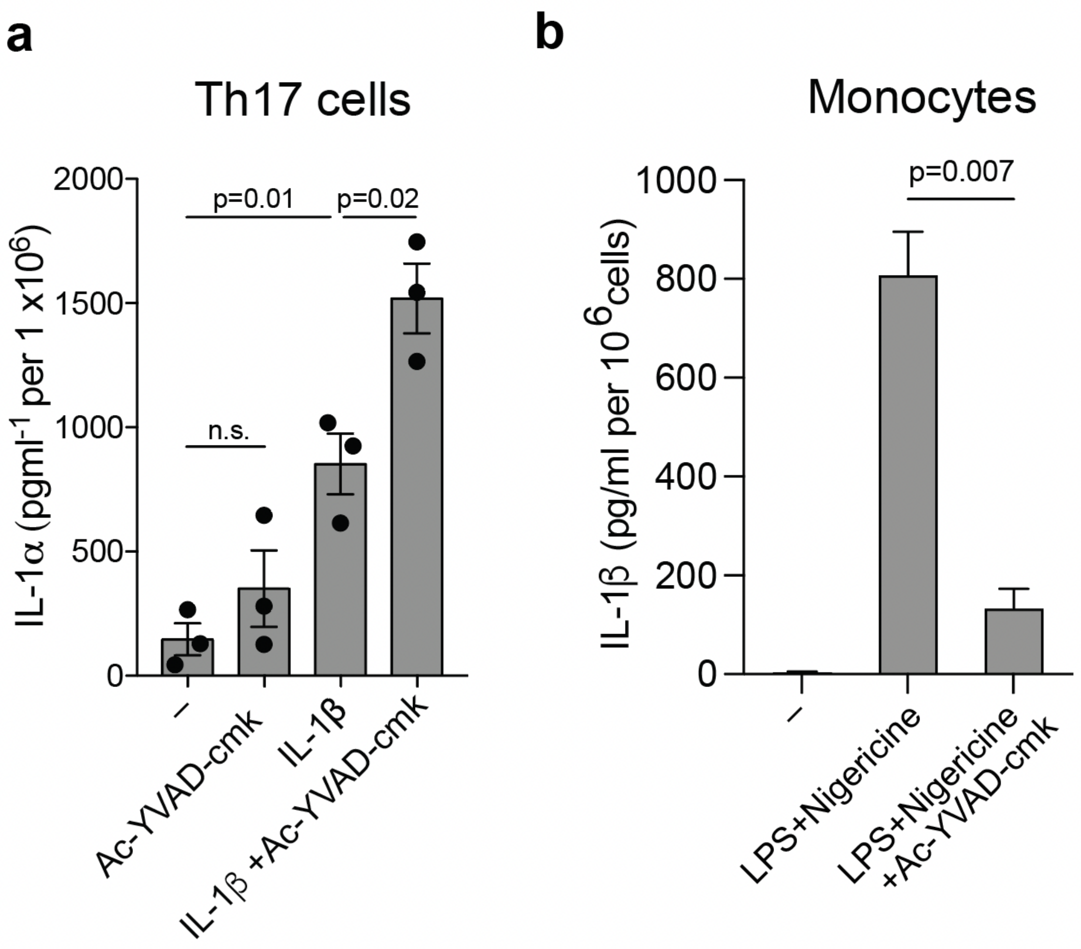

Supplementary Fig. 7. Selective caspase-1 inhibition does not abrogate IL-1α secretion by human Th17 cells but does abrogate IL-1β secretion in monocytes. a, ELISA in the presence or absence of the caspase-1 inhibitor Ac-YVAD-cmk without and with the IL-1**α** inducer IL-1**β**. Each circle indicates an individual healthy blood donor (n=3 biologically independent samples examined over 2 independent experiments). **b,** ELISA of IL-1**β** secretion by monocytes stimulated with LPS and nigericin in the absence or presence of Ac-YVAD-cmk. n=2 biologically independent samples examined over 2 independent experiments. One-way ANOVA. Data are presented as mean ± SEM.

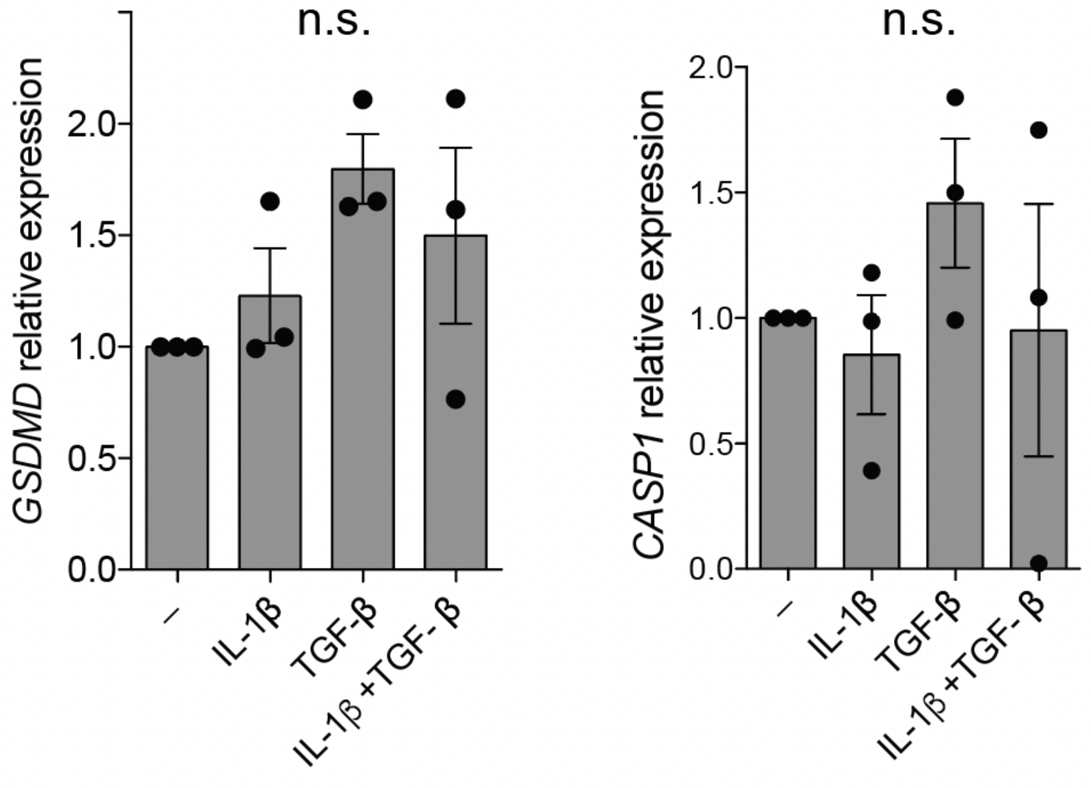

Supplementary Fig. 8. *GSDMD* and *CASP1* expression are not regulated by Th17-cell-polarizing cytokines. qRT–PCR analysis of Th17 cells that were stimulated for 5 days with anti-CD3 and anti-CD28 mAbs (48 h plate-bound) in the presence or absence of polarizing cytokines. One-way ANOVA. Each circle indicates an individual healthy blood donor (n=3 biologically independent samples examined over 2 independent experiments). Data are presented as mean ± SEM. n.s., not significant.

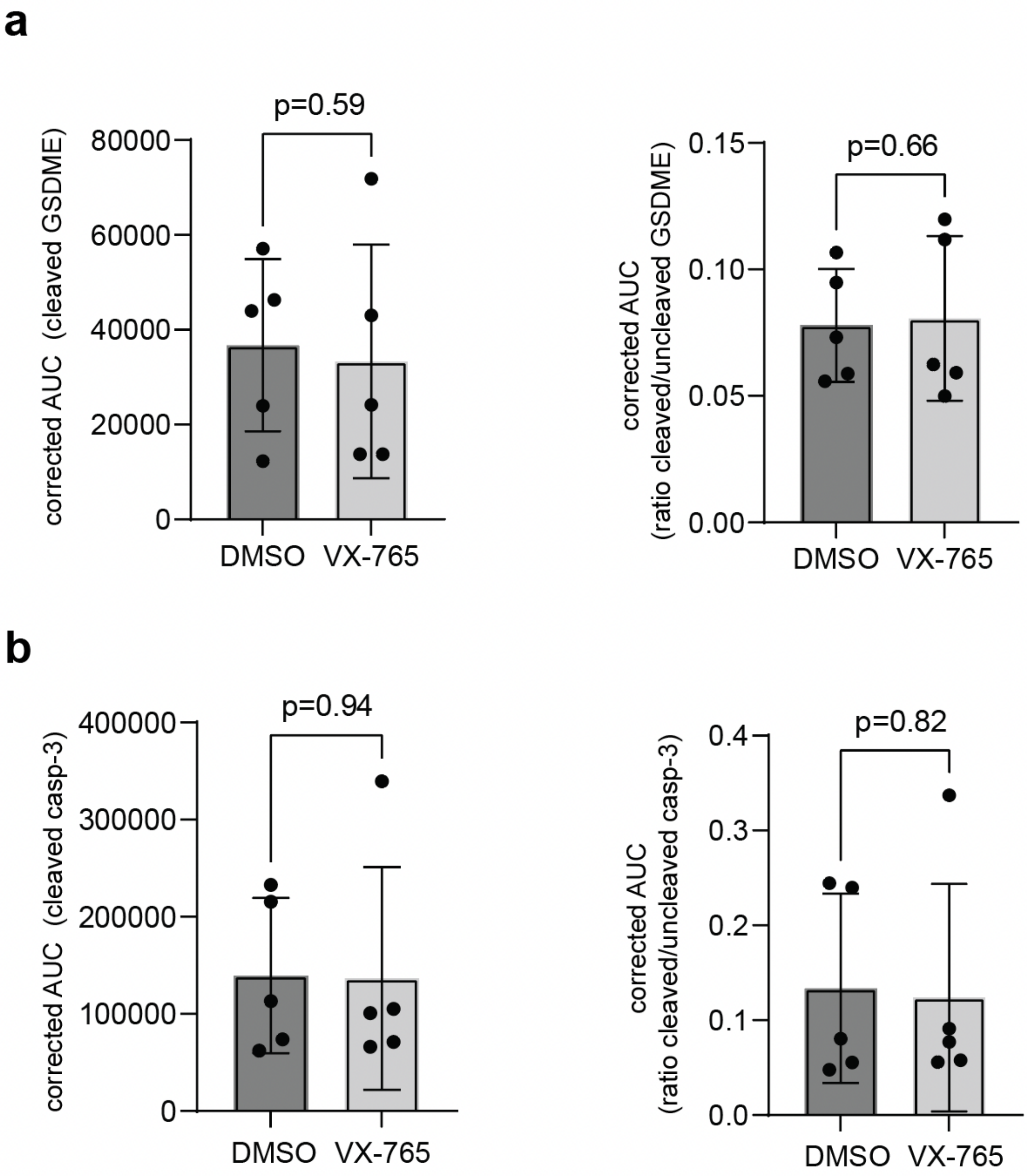

Supplementary Fig. 9. **Caspase-1 inhibition does not impact caspase-3 or GSDME cleavage. a, b,** Human Th17 cells were stimulated with anti-CD3 and anti-CD28 mAbs in the presence or absence of the caspase-1 inhibitor VX-765 for 5 days (inhibitor added on day 0 and day 3). The lysates were analysed by the Jess Simple Western System (ProteinSimple). The AUC was calculated with the Jess software Compass for SW as the ratio of the cleaved versus noncleaved inhibitor target proteins after normalization to total protein. Two-sided paired t-tests. Each circle indicates an individual healthy blood donor (n=5 biologically independent samples examined over 3 independent experiments). The data are presented as mean ± SEM.

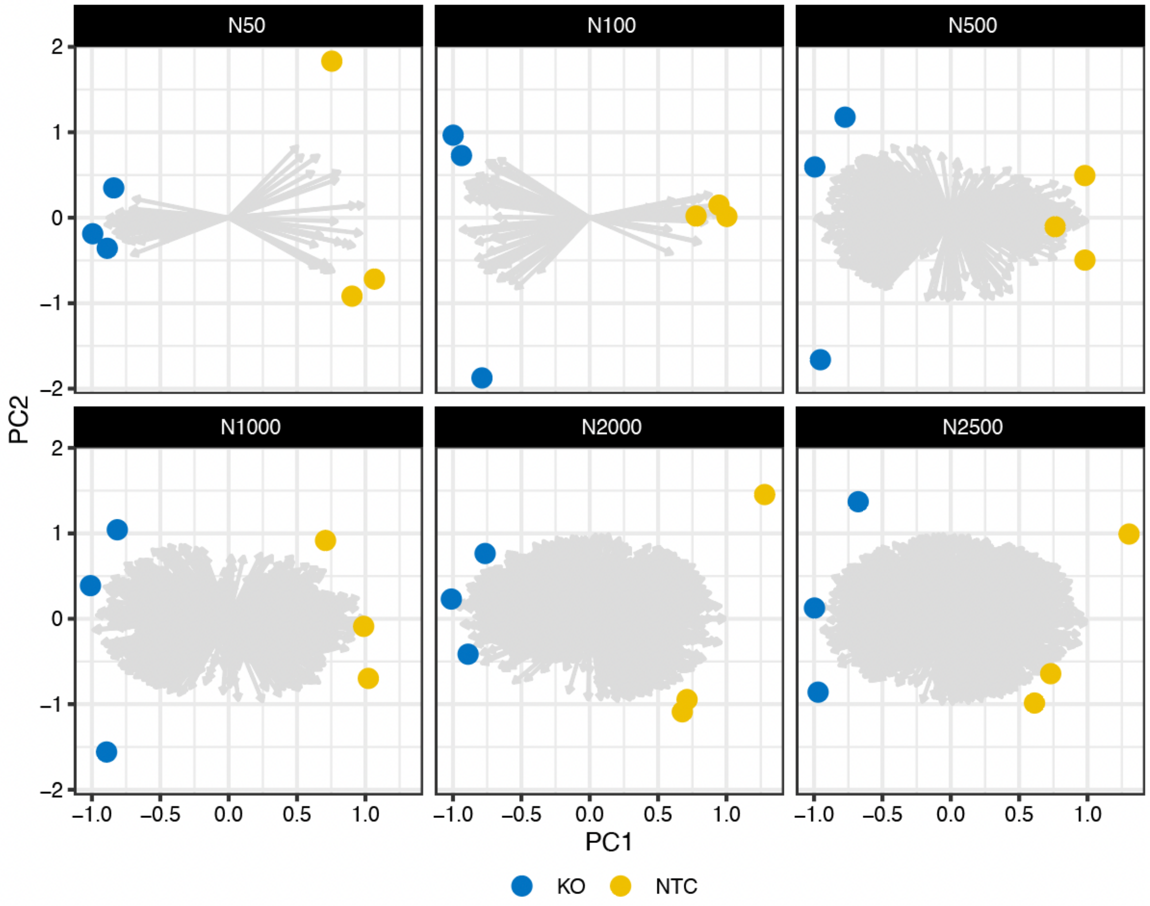

Supplementary Fig. 10. **GSDME expression by human Th17 cells is associated with transmembrane transport in the absence of cell death.** Transcriptomic comparison of GSDME intact (NTC) and CRISPR–Cas9-targeted GSDME-deficient (KO) human Th17 cells from three matched blood samples. Shown is the PCA biplot of the expression values of the genes with the N=50, 100, 500, 1000, 2000, or 2500 highest or lowest fold-changes (FCs) between the two groups of samples as the basis for the heatmap shown in Fig.7b. The KO and NTC samples (shown as filled circles) are clearly different from each other based on the first two principal components (PCs; PC1 and PC2). The arrows show the contribution of each gene to the PCs.

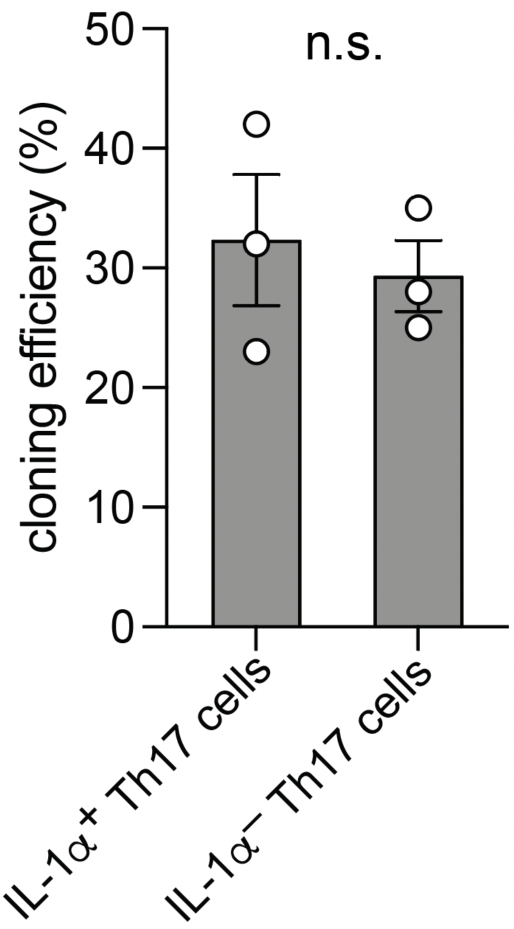

Supplementary Fig. 11. **Th17 cells that differ in IL-1α expression display the same single-cell cloning efficiency.** IL-1α$^+$ and IL-1α$^-$ Th17 cells were isolated with a homemade cytokine capture assay after stimulation of Th17 cells for 5 days with anti-CD3 and anti-CD28 mAbs. Single cells from both populations were then cloned by single-cell deposition and expansion with irradiated feeder cells, PHA and IL-2 (500 IU). Shown is the frequency of expanding T cells (clones) among seeded cells (cloning efficiency) among three independent blood donors and experiments shown as individual circles. n=3 biologically independent samples examined over 3 independent experiments. Two-sided paired t-test. Data are presented as mean ± SEM.
