# Supplementary material for: Human TH17 cells engage gasdermin E pores to release IL-1α on NLRP3 inflammasome activation
Source: Nat Immunol. 2023 Jan 5;24(2):295–308. doi: 10.1038/s41590-022-01386-w (PMC9892007; doi:10.1038/s41590-022-01386-w)
Supplement: Supplementary file 2 — Reporting Summary [file 41590_2022_1386_MOESM2_ESM.pdf]

nature portfolio

Corresponding author(s): Christina E. Zielinski

Last updated by author(s): 28.10.2022

Reporting Summary

Nature Portfolio wishes to improve the reproducibility of the work that we publish. This form provides structure for consistency and transparency in reporting. For further information on Nature Portfolio policies, see our Editorial Policies and the Editorial Policy Checklist.

Statistics

For all statistical analyses, confirm that the following items are present in the figure legend, table legend, main text, or Methods section.

n/a	Confirmed	
☐	☒	The exact sample size (*n*) for each experimental group/condition, given as a discrete number and unit of measurement
☐	☒	A statement on whether measurements were taken from distinct samples or whether the same sample was measured repeatedly
☐	☒	The statistical test(s) used AND whether they are one- or two-sided<br>*Only common tests should be described solely by name; describe more complex techniques in the Methods section.*
☒	☐	A description of all covariates tested
☒	☐	A description of any assumptions or corrections, such as tests of normality and adjustment for multiple comparisons
☐	☒	A full description of the statistical parameters including central tendency (e.g. means) or other basic estimates (e.g. regression coefficient) AND variation (e.g. standard deviation) or associated estimates of uncertainty (e.g. confidence intervals)
☐	☒	For null hypothesis testing, the test statistic (e.g. *F*, *t*, *r*) with confidence intervals, effect sizes, degrees of freedom and *P* value noted<br>*Give P values as exact values whenever suitable.*
☒	☐	For Bayesian analysis, information on the choice of priors and Markov chain Monte Carlo settings
☒	☐	For hierarchical and complex designs, identification of the appropriate level for tests and full reporting of outcomes
☒	☐	Estimates of effect sizes (e.g. Cohen's *d*, Pearson's *r*), indicating how they were calculated

Our web collection on statistics for biologists contains articles on many of the points above.

Software and code

Policy information about availability of computer code

Data collection	BD FACSAriaTM III (BD Biosciences), BD FACSAriaTM Fusion (BD Biosciences), BD LSRFortessa (BD Biosciences), CytoFLEX Flow Cytometer (Beckman Coulter), MACSQuant Analyzer (Miltenyi Biotec), ImageStream®X Mk II imaging flow cytometer (AMNIS®; MERCK Millipore), Odyssey Imaging system (LI-COR Biosciences), Jess System (ProteinSimple)
Data analysis	All the software and their version information, when available, are shown. FlowJo (always latest version up to 10.6.1 upon completion of the study) was used for FACS analyses. Scripts for bioinformatic analyses were written by Albert Garcia and Gianni Panagiotou (coauthors) and Sivia Fibi-Smetana and Leila Taher. Codes have been deposited publicly (Github and Zenodo) as indicated in the manuscript (Code availability statement). GraphPad Prism (v.7-9) was used to analyzye data and to create plots. INSPIRE software, IDEAS software 6.2.64.0, Image Studio™ Lite (LI-COR Biosciences) 5.0, Compass software 6.0.0 (ProteinSimple), R version 4.1.

For manuscripts utilizing custom algorithms or software that are central to the research but not yet described in published literature, software must be made available to editors and reviewers. We strongly encourage code deposition in a community repository (e.g. GitHub). See the Nature Portfolio guidelines for submitting code & software for further information.

March 2021

Data

Policy information about availability of data

All manuscripts must include a data availability statement. This statement should provide the following information, where applicable:

- Accession codes, unique identifiers, or web links for publicly available datasets
- A description of any restrictions on data availability
- For clinical datasets or third party data, please ensure that the statement adheres to our policy

Raw and processed Sequencing Data files are available GEO. All accession numbers are provided in the manuscript. All data points for the remaining experiments are shown in the paper. All data points prepresent individual biological samples as indicated in the legends.

Field-specific reporting

Please select the one below that is the best fit for your research. If you are not sure, read the appropriate sections before making your selection.

☒ Life sciences ☐ Behavioural & social sciences ☐ Ecological, evolutionary & environmental sciences

For a reference copy of the document with all sections, see nature.com/documents/nr-reporting-summary-flat.pdf

Life sciences study design

All studies must disclose on these points even when the disclosure is negative.

Sample size	The sample sizes are indicated in the respective figures with circles (in bar graphs) indicating individual donors and experiments. Sample sizes were based on our experience and common practice in the field of human immunology (i.e. Nat Immunol. 2018 Oct; 19(10): 1126–1136.)
Data exclusions	no data exclusions
Replication	Each data point indicates an independent blood donor. Multiple blood donors and experiments were performed to confirm the conclusions. The individual data points, which correlate with independent blood donors are shown in the respective graphs.
Randomization	Healthy donor blood from men and women (anonymous) was used. Patient samples (JIA)were provided solely based on the diagnosis.
Blinding	Blinding was not relevant for this study. For JIA patients blood collection, experiment and data analysis were done by three independent groups, respectively.

Reporting for specific materials, systems and methods

We require information from authors about some types of materials, experimental systems and methods used in many studies. Here, indicate whether each material, system or method listed is relevant to your study. If you are not sure if a list item applies to your research, read the appropriate section before selecting a response.

Materials & experimental systems

n/a	Involved in the study	
☐	☒	Antibodies
☐	☒	Eukaryotic cell lines
☒	☐	Palaeontology and archaeology
☒	☐	Animals and other organisms
☐	☒	Human research participants
☒	☐	Clinical data
☒	☐	Dual use research of concern

Methods

n/a	Involved in the study	
☒	☐	ChIP-seq
☐	☒	Flow cytometry
☒	☐	MRI-based neuroimaging

Antibodies

Antibodies used

Antigen; conjugate (if applicable); dilution; clone; vendor; Order number
FACS/Imaging flow cytometry
ASC; PE; 1:50; HASC-71; Biolegend; 653904
CCR4; PE/Cy7; 1:200; L291H4; Biolegend; 359410
CCR6; PE; 1:50; 11A9; BD; 559562
CD14; PacificBlue; 1:200-1:400; HCD14; Biolegend; 325616
CD3; FITC; 1:150; UCHT1; Biolegend; 300440
CD3; APC; 1:100; UCHT1; Biolegend; 300412

CD45RA; FITC; 1:200; HI100; Biolegend; 304106
CD8; PacificBlue; 1:100; SK1; Biolegend; 344718
CXCR3; APC; 1:10; 1C6/CXCR3; BD; 550967
IFN-γ; APC/Cy7; 1:300; 4S.B3; Biolegend; 502530
IL-10; PE/Cy7; 1:50; JES3-9D7; Biolegend; 501420
IL-10; APC; 1:50; JES3-9D7; BD ; 554707
IL-10; PE; 1:10; JES3-9D7; BD; 559330
IL-17A; PacificBlue; 1:100; BL168; Biolegend; 512312
IL-1a; PE; 1:50; 364-3B3-14; Biolegend; 500106
IL-1R1; PE; 1:20; FAB269P; R&D; FAB269P-100
IL-4; FITC; 1:600; MP4-25D2; Biolegend; 500807
Ki-67; Brilliant Violet 421; 1:10; Ki-67; Biolegend; 350506
NALP3/NLRP3; APC; 1:50; REA668; Miltenyi; 130-111-210
RORγt; APC; 1:10; AFKJS-9; eBioscience; 17-6988-82
IL-1b; Alexa Fluor 647; 1:50; JK1B-1; Biolegend; 508207
CCR7; PE;1:50; G043H7; Biolegend;353203
CD25; BV421;1:100;BC96; Biolegend;302640
Western blot /Jess
caspase 8; 1:50 (Jess) 1:1000 (WB); 1C12; Cell signaling; 9746T;
caspase 1; 1:1000 (WB); polyclonal; Cell signaling; 2225S
b-actin; 1:2000 (WB) 1:200 (Jess); 8H10D10; Cell signaling; 3700S
caspase-3; 1:1000 (WB) 1:50 (Jess); polyclonal; Cell signaling; 9662
gasdermin D; 1:1000 (WB) 1:50 (Jess); polyclonal; Cell signaling; 96458
cleaved gasdermin D; 1:50 (Jess) 1:1000(WB); E7H9G; Cell signaling; 36425S
NLRP3; 1:2000 (WB); D2P5E; Cell signaling; 13158S
Mouse IgG; HRP; 1:2000 (WB); polyclonal; Cell signaling; 7076
Rabbit IgG; HRP; 1:2000 (WB); polyclonal; Cell signaling; 7074
IL-1α; 1:1000 (WB); EPR5103(2); Abcam; ab134908
gasdermin E; 1:50 (Jess) 1:500 (WB); EPR19859; Abcam; ab215191;
Sodium Potassium ATPase; 1:50 (Jess); EP1845Y; Abcam; ab76020
GAPDH; 1:1000 (WB) 1:100 (Jess); 6C5; MERCK; CB1001
ASC; 1:50 (Jess); B-3; Santa Cruz Biotechnology; sc-514414
NLRP3/NALP3; 1:50 (Jess); 25N10E9; Novus Biologicals; NBP2-03948;
Anti mouse detection module; HRP; as per manufacturer`s instructions; Protein Simple; DM-002
Anti rabbit detection module; HRP; as per manufacturer`s instructions; Protein Simple; DM-001

Validation

FACS antibodies validation:

Biolegend - https://www.biolegend.com/en-us/quality/quality-control

BD - https://www.biocompare.com/Antibody-Manufacturing/355107-Antibody-Manufacturing-Perspectives-BD-Bioscience/

Milteniy Biotec - https://www.miltenyibiotec.com/DE-en/products/macs-antibodies/antibody-validation.html#gref

All FACS antibodies are commercially available and verifications can be found on respective manufacturer's website.
All antibodies have in addition been tested on the cells used herein by performing titrations according to standard recommendations (Eur J Immunol. 2019 Oct;49(10):1457-1973. doi: 10.1002/eji.201970107). They were then used at the concentrations indicated herein and in the methods section of the manuscript.

Western blot/Jess antibodies validation:

Cell Signaling Technology - https://www.cellsignal.de/about-us/our-approach-process/antibody-validation-western-blotting

Abcam - https://www.abcam.com/primary-antibodies/how-we-validate-our-antibodies#Western%20blot

NOVUS Biologicals - https://www.novusbio.com/5-pillars-validation

Santa Cruz Biotechnology - https://www.labome.com/method/Santa-Cruz-Antibodies.html

MERCK - https://www.sigmaaldrich.com/DE/en/technical-documents/technical-article/protein-biology/immunohistochemistry/antibody-enhanced-validation

All antibodies have in addition been tested on the cells used herein by performing titrations according to standard recommendations (Eur J Immunol. 2019 Oct;49(10):1457-1973. doi: 10.1002/eji.201970107). They were then used at the concentrations indicated herein and in the methods section of the manuscript.

All western blot antibodies are commercially available and verifications can be found on respective manufacturer's website. In addition, western blot antibodies were validated using genetic strategy: expression of the target protein was compared before and after knockout using CRISPR/Cas9 technology. If protein expression following knockout was substantially reduced, then antibody was considered as specific.

Eukaryotic cell lines

Policy information about cell lines

Cell line source(s)	Allogeneic PBMCs were used as feeder cells and were isolated from healthy donors. T cell lines and T cell clones were generated from primary human cells and kept short term in culture.
Authentication	does not apply
Mycoplasma contamination	not tested in primary human t cells.
Commonly misidentified lines (See ICLAC register)	does not apply

Human research participants

Policy information about studies involving human research participants

Population characteristics	healthy, men and women, age: 22-65
Recruitment	fresh blood from healthy anonymous blood donors and buffy coats from the blood banks of the Charite Universitätsmedizin Berlin and the Universitätsklinikum Jena were used whenever needed. Clinical blood and synovial fluid samples were obtained from Bas Vastert (University Medical Center Utrecht, Biobank). The recruitment occured based on diagnosis and the samples were stored in a biobank and selected randomly based on the diagnosis criterium only.
Ethics oversight	The ethics committees of the Charité Universitätsmedizin Berlin, the Technical University of Munich and the Friedrich Schiller University of Jena approved the study with with positive ethics votes.

Note that full information on the approval of the study protocol must also be provided in the manuscript.

Flow Cytometry

Plots

Confirm that:

☒ The axis labels state the marker and fluorochrome used (e.g. CD4-FITC).

☒ The axis scales are clearly visible. Include numbers along axes only for bottom left plot of group (a 'group' is an analysis of identical markers).

☒ All plots are contour plots with outliers or pseudocolor plots.

☒ A numerical value for number of cells or percentage (with statistics) is provided.

Methodology

Sample preparation	Primary cells were isolated as described in the methods (Ficoll isolation, positive magnetic isolation using microbeads, flow-cytometry assisted cell sorting)
Instrument	BD FACSAria, BD LSRFortessa, Cytoflex, Cytex AuroraBD, FACSAriaTM III (BD Biosciences), BD FACSAriaTM Fusion (BD Biosciences), BD LSRFortessa (BD Biosciences), CytoFLEX Flow Cytometer (Beckman Coulter), MACSQuant Analyzer (Miltenyi Biotec)
Software	FlowJo Software (Tree Star Inc) for FACS analyses
Cell population abundance	Purity of the relevant cell populations was checked after sorting and found to be >98%
Gating strategy	The gating strategies are shown in the Extended Data Fig. File 3. Lymphocytes were gated by FSC/SSC and exclusion of dead cells by zombie dye, exlcusion of doublets as shown, and further gating for CD4+CD14– CD3+ T cells and subgating for memory marker CD45RA– (CD45RA– for memory T cells, CD45RA+ for naive T cells) and the differential expression of chemokine receptors for the respective T helper cell subsets as shown and explained in the methods and the results section. The positive populations were defined with the use of unstained and single-stained controls and normally were above 10^3 on a log scale.

☒ Tick this box to confirm that a figure exemplifying the gating strategy is provided in the Supplementary Information.
