# Supplementary material for: Human TH17 cells engage gasdermin E pores to release IL-1α on NLRP3 inflammasome activation
Source: Nat Immunol. 2023 Jan 5;24(2):295–308. doi: 10.1038/s41590-022-01386-w (PMC9892007; doi:10.1038/s41590-022-01386-w)
Supplement: Supplementary file 4 — Unprocessed immunoblot scans. [file 41590_2022_1386_MOESM4_ESM.pdf]

Supplementary Fig.5

Supplementary Fig.5a

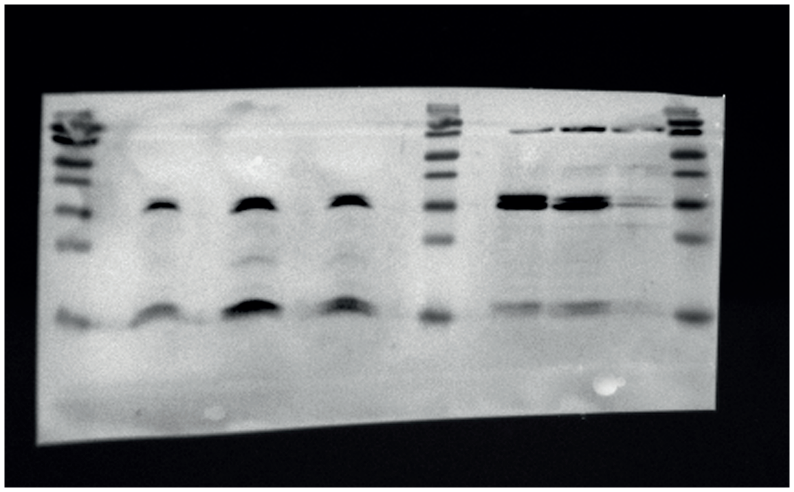

anti-IL-1a

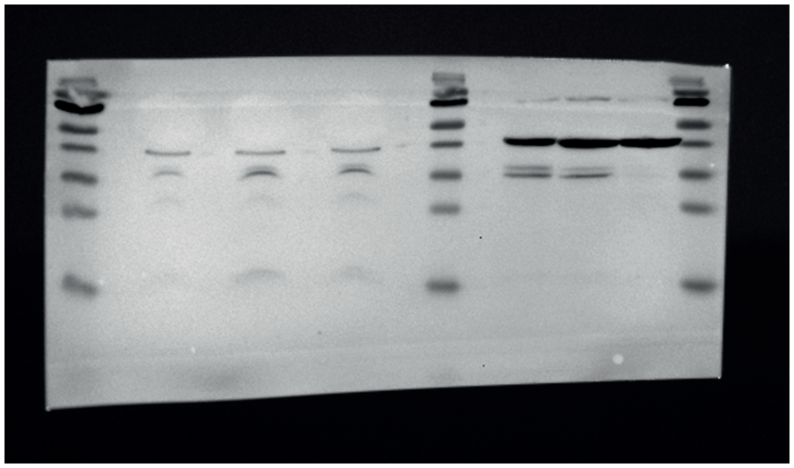

anti-b-actin

Supplementary Fig.5b

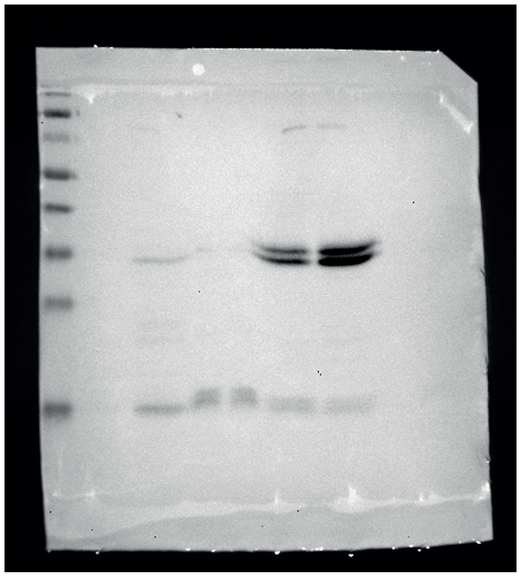

anti-IL-1a

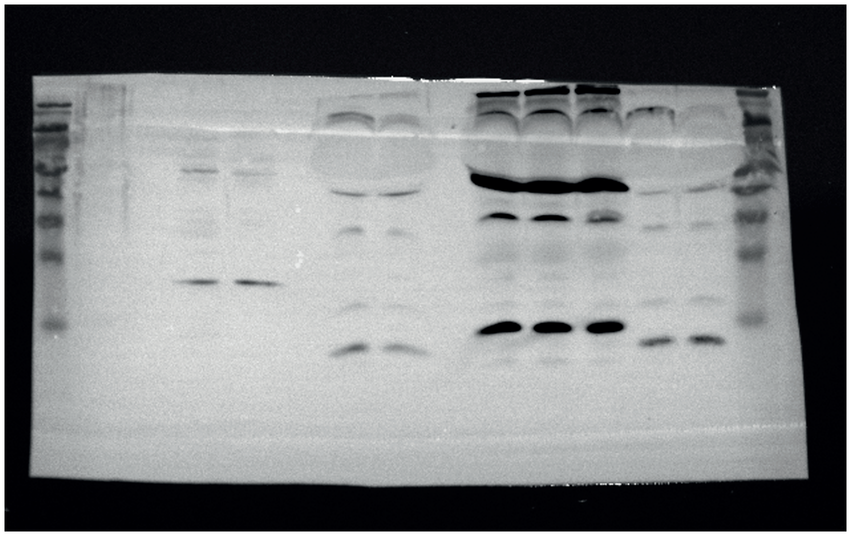

anti-IL-1a
