# Supplementary figures and images for: Human TH17 cells engage gasdermin E pores to release IL-1α on NLRP3 inflammasome activation
Source: Nat Immunol. 2023 Jan 5;24(2):295–308. doi: 10.1038/s41590-022-01386-w (PMC9892007; doi:10.1038/s41590-022-01386-w)

Fig. 4

Fig. 4a

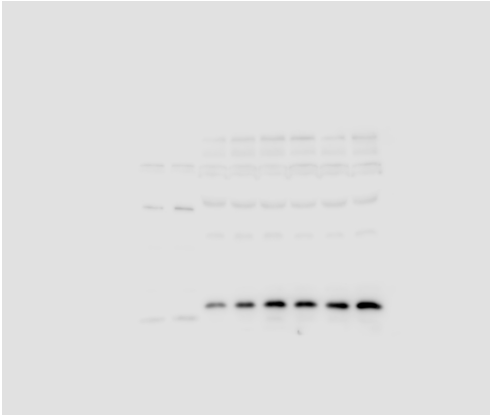

clone lysates

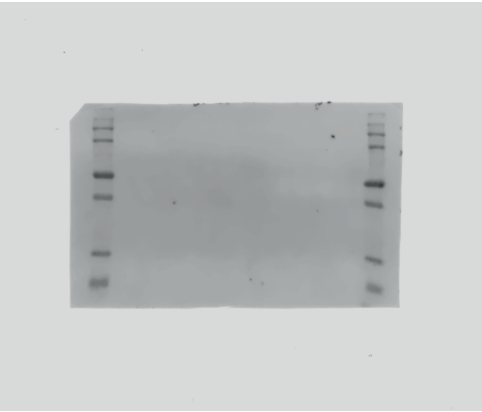

ladder

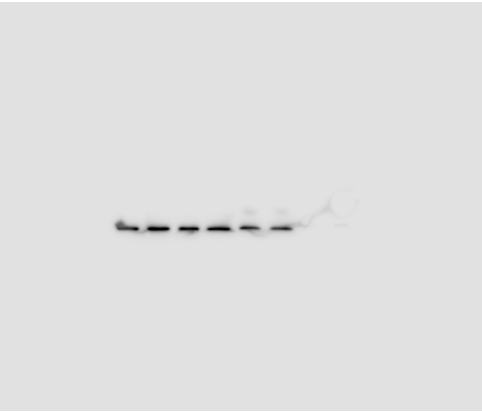

GAPDH

Fig. 4d

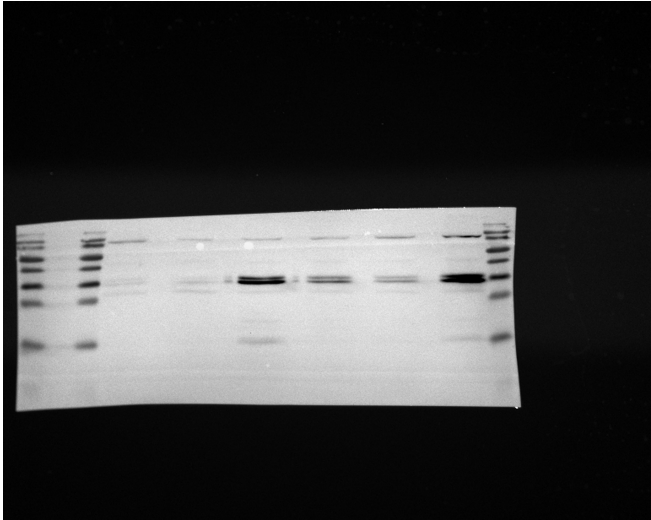

anti-IL-1a

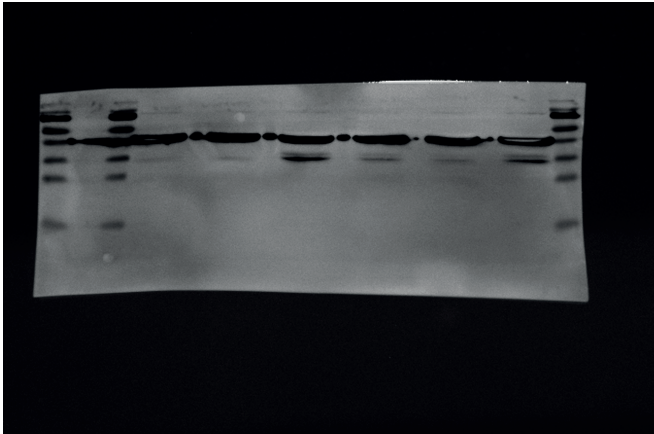

b-actin

Supplement: Source Data Fig. 4 — Unprocessed immunoblot. [file 41590_2022_1386_MOESM13_ESM.pdf]

High exposure

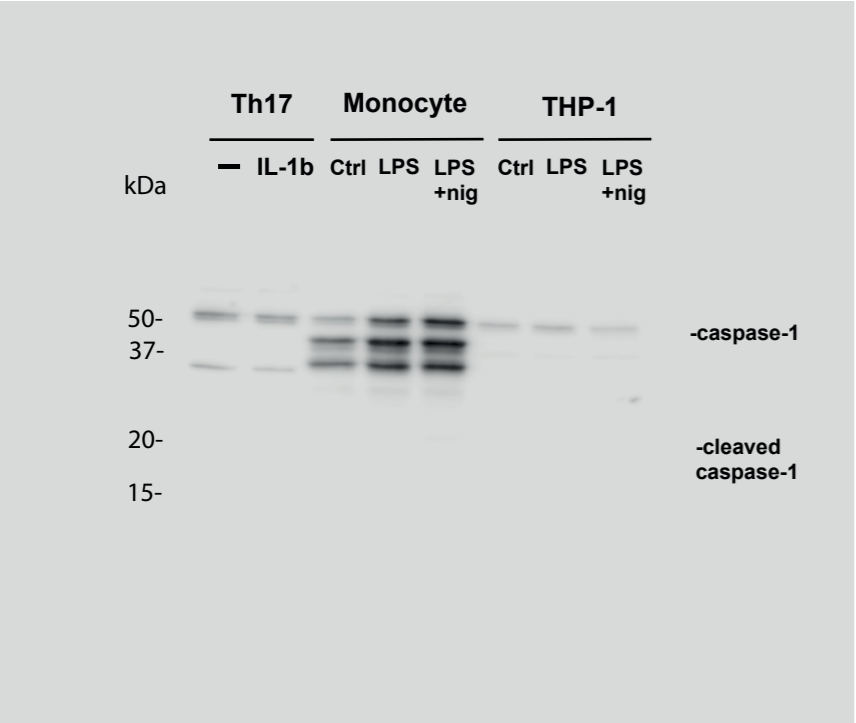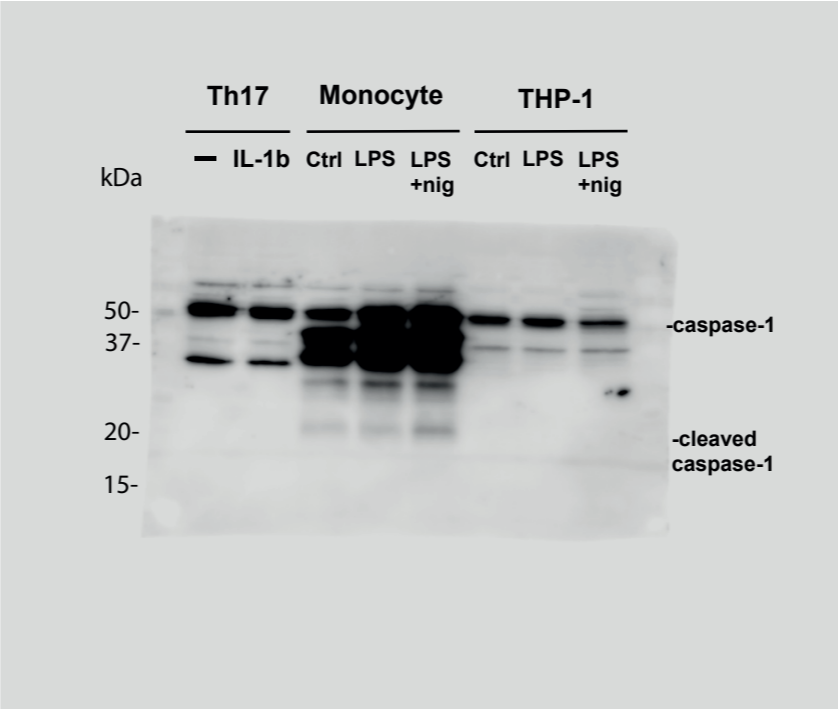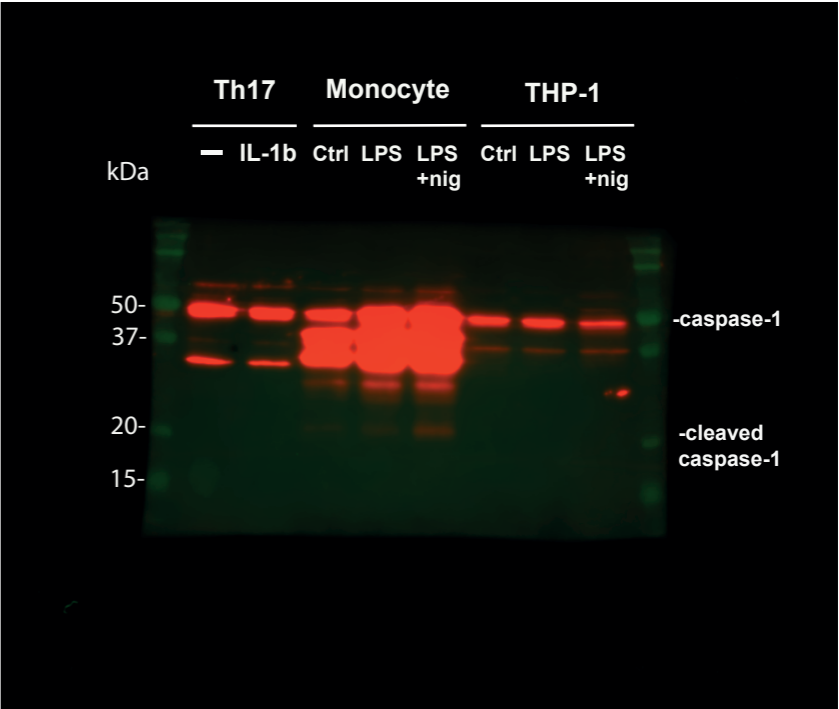

GAPDH

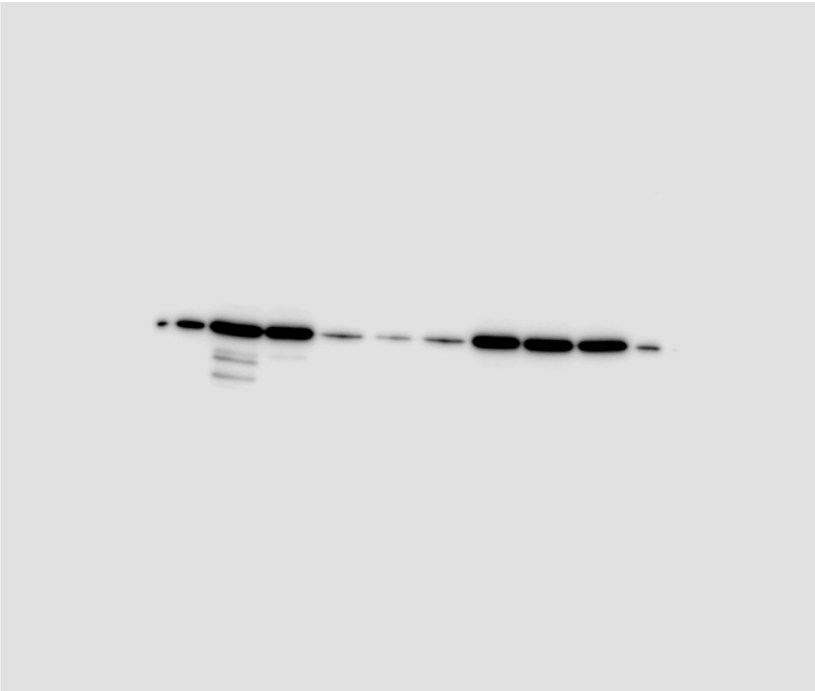

Supplement: Source Data Fig. 5 — Unprocessed immunoblot. [file 41590_2022_1386_MOESM14_ESM.pdf]

anti-NLRP3

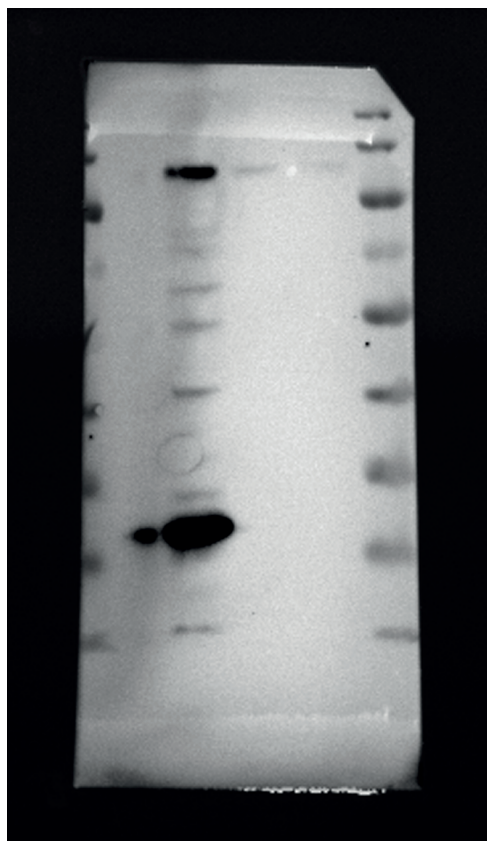

anti-NLRP3  
high exposure

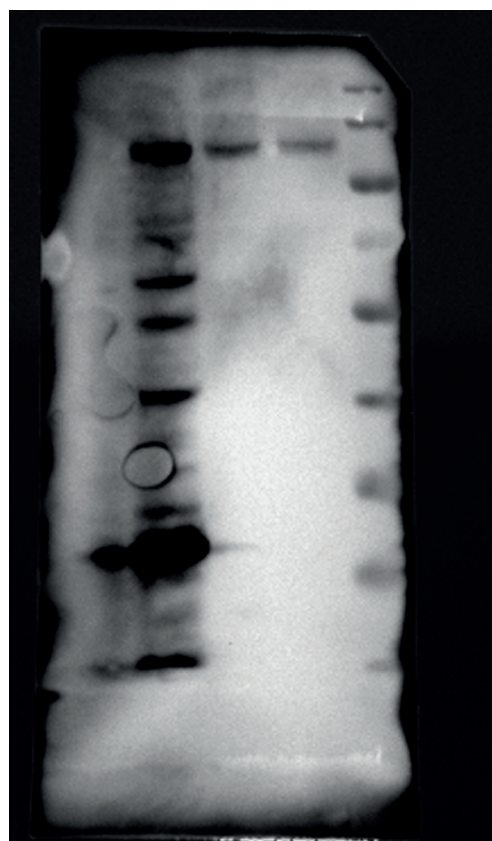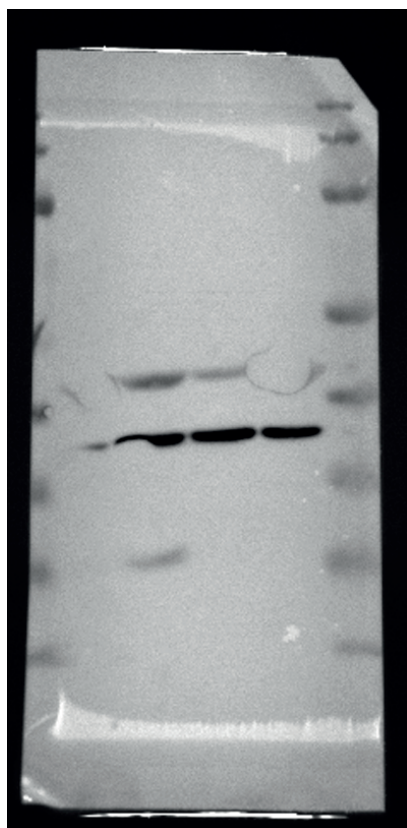

anti-GAPDH

Supplement: Source Data Extended Fig. 6 — Unprocessed immunoblot. [file 41590_2022_1386_MOESM18_ESM.pdf]
