# Supplementary material for: Human TH17 cells engage gasdermin E pores to release IL-1α on NLRP3 inflammasome activation
Source: Nat Immunol. 2023 Jan 5;24(2):295–308. doi: 10.1038/s41590-022-01386-w (PMC9892007; doi:10.1038/s41590-022-01386-w)
Supplement: Source Data Fig. 6 — Unprocessed immunoblot. [file 41590_2022_1386_MOESM15_ESM.pdf]

Fig.6

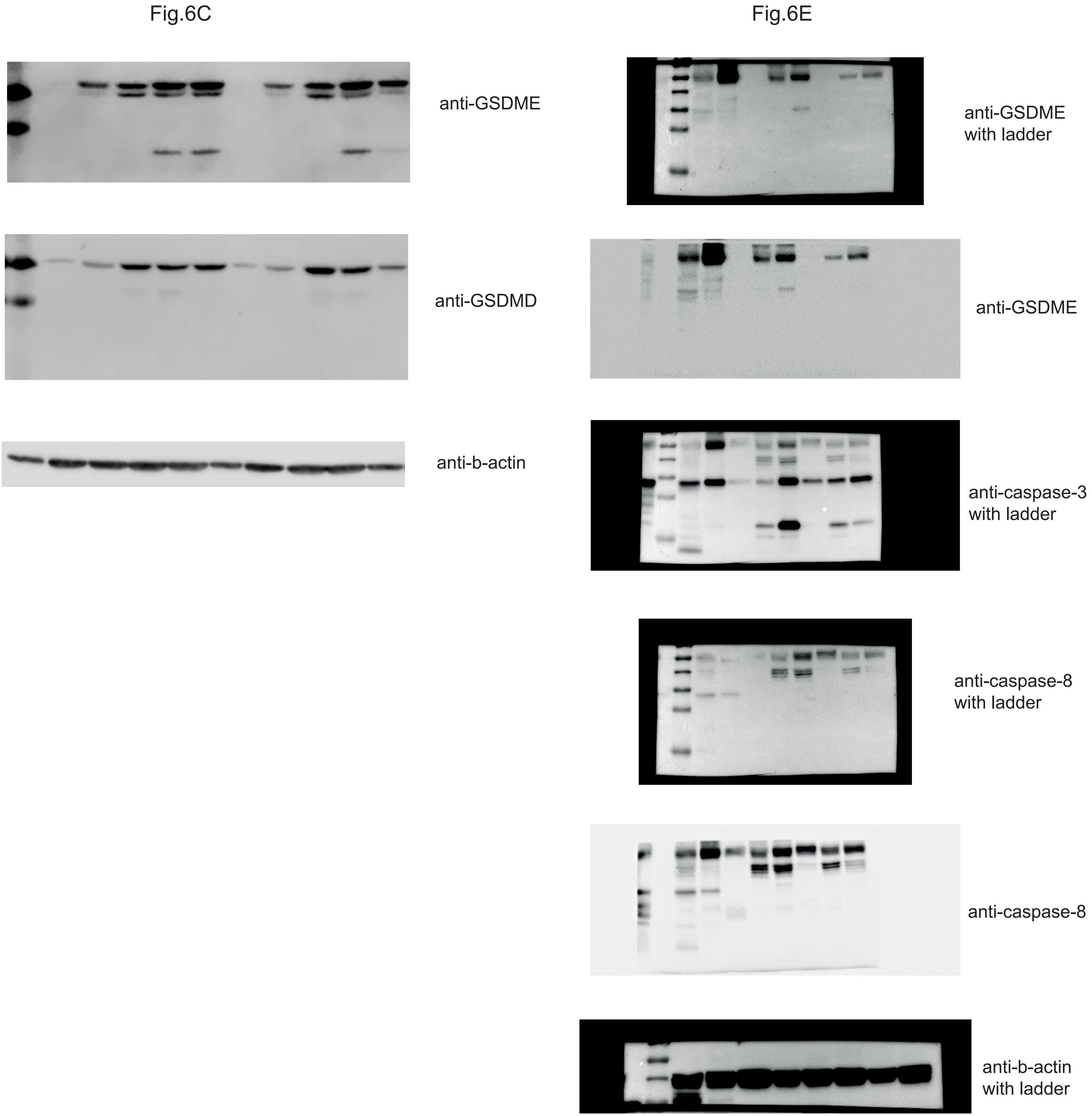

Fig.6C
anti-GSDME
anti-GSDMD
anti-b-actin
Fig.6E
anti-GSDME with ladder
anti-GSDME
anti-caspase-3 with ladder
anti-caspase-8 with ladder
anti-caspase-8
anti-b-actin with ladder
